# Bioprocessing of Mesenchymal Stem Cells and Their Derivatives: Toward Cell-Free Therapeutics

**DOI:** 10.1155/2018/9415367

**Published:** 2018-09-12

**Authors:** Jolene Phelps, Amir Sanati-Nezhad, Mark Ungrin, Neil A. Duncan, Arindom Sen

**Affiliations:** ^1^Pharmaceutical Production Research Facility, Department of Chemical and Petroleum Engineering, Schulich School of Engineering, University of Calgary, 2500 University Drive N.W., Calgary, AB, Canada T2N 1N4; ^2^Biomedical Engineering Graduate Program, University of Calgary, 2500 University Drive N.W., Calgary, AB, Canada T2N 1N4; ^3^BioMEMS and Bioinspired Microfluidic Laboratory, Department of Mechanical and Manufacturing Engineering, Schulich School of Engineering, University of Calgary, 2500 University Drive N.W., Calgary, AB, Canada T2N 1N4; ^4^Center for Bioengineering Research and Education, Schulich School of Engineering, University of Calgary, 2500 University Drive N.W., Calgary, AB, Canada T2N 1N4; ^5^Faculty of Veterinary Medicine, Heritage Medical Research Building, University of Calgary, 3330 Hospital Drive N.W., Calgary, AB, Canada T2N 4N1; ^6^Musculoskeletal Mechanobiology and Multiscale Mechanics Bioengineering Lab, Department of Civil Engineering, Schulich School of Engineering, University of Calgary, 2500 University Drive N.W., Calgary, AB, Canada T2N 1N4

## Abstract

Mesenchymal stem cells (MSCs) have attracted tremendous research interest due to their ability to repair tissues and reduce inflammation when implanted into a damaged or diseased site. These therapeutic effects have been largely attributed to the collection of biomolecules they secrete (i.e., their secretome). Recent studies have provided evidence that similar effects may be produced by utilizing only the secretome fraction containing extracellular vesicles (EVs). EVs are cell-derived, membrane-bound vesicles that contain various biomolecules. Due to their small size and relative mobility, they provide a stable mechanism to deliver biomolecules (i.e., biological signals) throughout an organism. The use of the MSC secretome, or its components, has advantages over the implantation of the MSCs themselves: (i) signals can be bioengineered and scaled to specific dosages, and (ii) the nonliving nature of the secretome enables it to be efficiently stored and transported. However, since the composition and therapeutic benefit of the secretome can be influenced by cell source, culture conditions, isolation methods, and storage conditions, there is a need for standardization of bioprocessing parameters. This review focuses on key parameters within the MSC culture environment that affect the nature and functionality of the secretome. This information is pertinent to the development of bioprocesses aimed at scaling up the production of secretome-derived products for their use as therapeutics.

## 1. Introduction

Mesenchymal stem cells (MSCs) are unspecialized cells that can be isolated from various tissues within the body including bone marrow, adipose, dermal, umbilical cord blood, and synovial fluid [1–3]. A cell population isolated from these tissues is considered to contain primarily MSCs if it meets the following minimum criteria defined by the International Society for Cellular Therapy: (i) the cell population must be plastic-adherent; (ii) ≥95% of the cell population needs to express the surface antigens CD105, CD73, and CD90 and ≤2% may express CD45, CD34, CD14 or CD11b, CD79*α* or CD19, and HLA-DR; and (iii) the cells need to be able to differentiate to bone, fat, and cartilage fates *in vitro* [4].

MSCs have attracted great research interest for the treatment of medical disorders due to their ability to repair tissues and reduce inflammation when implanted into a damaged or diseased site. Numerous clinical trials have now demonstrated the safety and feasibility of MSC implantation therapies in applications of tissue repair, as well as in disease mitigation through immunomodulation [5]. However, despite moderate successes, many concerns remain regarding the therapeutic efficacy of MSCs due to the high degree of variability in clinical outcomes [6]. There is a clear need to find methods that can consistently yield positive results. MSC therapies also face challenges in having to immunologically match donors and recipients to minimize the possibility of rejection, as well as technical considerations around the storage and transport of viable cells. Furthermore, in many cases it has been found that there is very limited retention of MSCs within an injury site. Despite reports of therapeutic benefits, often less than 1% of the transplanted MSCs are retained long-term within the target tissue [7, 8].

Whereas it was initially believed that these cells contribute to tissue repair by differentiating into the specialized cell types required to replace the dead and damaged cells native to that tissue, there is increasing evidence to suggest that much of the observed therapeutic benefit associated with MSC therapy may be attributed to the bioactivity of factors and molecules secreted by these cells. In fact, the focus of many clinical trials has been to evaluate the therapeutic effects of the factors and molecules produced by mesenchymal stem cells, rather than integration of the cells themselves. These secreted factors and molecules, collectively referred to as the MSC “secretome,” are hypothesized to upregulate endogenous repair and immunomodulation mechanisms [9]. It has even been proposed that MSCs now be referred to as medicinal signalling cells to more accurately reflect their mode of action [10]. This raises the possibility of administering MSC-derived products as therapeutics rather than implanting the cells themselves, which would address some of the key challenges for the clinical translation of MSC-based therapies.

Registered clinical trials are currently underway to evaluate the effectiveness of extracellular vesicles derived from the MSC secretome, including one involving patients with ischemic stroke (December 2017), a second for the healing of macular holes (February 2018), and a third involving the maintenance of *β*-cell mass in type I diabetes mellitus (T1DM) (2014) [11]. Prior studies utilizing MSC-derived extracellular vesicles in human patients for graft versus host disease (GvHD) [12] and chronic kidney disease (CKD) [13], demonstrated improved outcomes and immunosuppressive effects. The exclusion of implanted cells in this approach means products can be bioengineered to enhance therapeutic potential and improve quality control, can be scaled to specific dosages, and benefits from reduced immunogenicity [14]. In addition, the nonliving nature of the secretome means that it can be characterized, stored, packaged, and transported significantly more easily than viable cells—a critical consideration for the economic viability of new therapies.

Several challenges need to be overcome to make this technology clinically available and to utilize the MSC secretome as a cell-free therapeutic. The MSC secretome differs depending on the tissue from which the MSCs are isolated, and there is substantial variation between donors and in response to differing culture conditions [15, 16]. While much work has been done to understand how the cells themselves change in response to environmental factors such as oxygenation, mechanical forces, and chemical stimuli, considerably less work has focused on the effect of these factors on resultant secretome profiles. Such studies would not only enable secretome optimization for specific applications but also provide an essential foundation for larger-scale production. Though it is simple and cost-effective to study MSCs in static monolayer cultures, such conditions are not conducive to large-scale production. This review outlines the therapeutic products that can be obtained from MSCs and important culture parameters that need to be considered for the scalable production and clinical translation of the MSC secretome.

## 2. The Composition of the MSC Secretome

The MSC secretome contains many cell signalling molecules, including growth factors and cytokines that modulate cell behaviours such as proliferation, differentiation, and extracellular matrix production or provide pro- and anti-inflammatory effects. Recent studies have provided evidence that MSCs also secrete small membrane-bound extracellular vesicles (EVs) that contain a number of biomolecules, including not only growth factors and cytokines but also various forms of RNA capable of triggering a variety of biological responses throughout an organism [17]. Notably, it has recently been reported that EVs alone may provide similar or enhanced therapeutic benefit to their cellular counterparts [18].

During a culture period, the MSC secretome can be recovered from the expended medium. The term “conditioned medium” (CM) is used to describe an expended medium, or a combination of fresh medium and expended medium from prior cell cultures. CM is primarily prepared by centrifuging expended medium to remove cell debris and then using the resulting supernatant directly, or by adding a concentrated or fractionated form of it to fresh medium. By fractionating the CM, it is possible to correlate a particular molecular subset with a specific measured effect. Studies spanning a wide array of physiological applications have demonstrated the benefits of the MSC secretome through the utilization of CM.  Table 1 outlines the biological effects identified from the MSC secretome or MSC secretome-derived products, in various disease models.

### 2.1. Cytokines and Growth Factors

MSCs secrete a wide variety of cell signalling cytokines and growth factors. These bioactive molecules can stimulate endogenous cell populations to undergo responses which may contribute to healing in a variety of tissues. Some of the most physiologically relevant biomolecules secreted by MSCs include hepatocyte growth factor (HGF), which has been reported to be involved in immunomodulation, cell migration, development, wound healing and antiapoptosis; transforming growth factor- (TGF-) *β* potentiated in immunomodulation, cell growth, proliferation and differentiation, and wound healing; vascular endothelial growth factor (VEGF), playing a large role in angiogenesis but also in immunomodulation and cell survival; and molecules such as tumor necrosis factor-stimulated gene- (TSG-) 6, prostaglandin E2 (PGE2), and galectins 1 and 9 which are all reported to play a large role in immunomodulation [19, 20]. For a more detailed examination, see the thorough review by Bai et al. [19] that describes the function of bioactive molecules secreted by umbilical cord-derived MSCs.

Various clinical trials have injected individual biomolecular species in an effort to elicit a positive therapeutic response [21–23]. The injection of vascular endothelial growth factor (VEGF) was effective in improving angiogenesis in coronary heart disease patients; however, such trials have not been able to match the therapeutic efficacy of MSCs [24]. Similarly, high-dose bolus interleukin-2 (IL-2) has FDA approval for metastatic melanoma and renal cell carcinoma, but is challenged by low response rates and notorious toxicities [23].

CM derived from MSC cultures has shown promising benefits in a wide range of therapeutic and immunomodulatory applications, including the treatment of skin wounds, distraction osteogenesis, and kidney injury [25–28]. Despite the wide array of bioactive molecules released by MSCs, their use as a therapeutic is limited by their stability. Under physiological conditions, the functional stability of cytokines and growth factors can decay within minutes [29]. It has been shown that many of these same bioactive molecules can also be found within the EVs secreted by MSCs, albeit in much lower amounts [30, 31]. Remarkably, it has been demonstrated that EVs, but not EV-depleted CM, can elicit therapeutic benefits such as rescuing the retardation of fracture healing in a CD9^−/−^ mouse model [31]. This suggests that the bioactivity possessed by EVs may give clinical value to such secreted structures.

### 2.2. Extracellular Vesicles

EVs are phospholipid membrane-bound particles secreted from cells that contain biological materials including DNA, RNA, bioactive lipids, and proteins. The internal components, or “cargo,” are specific to cell source (i.e., the individual, as well as the particular tissue from which the MSCs were derived) and the pathological state of the cells. EVs can be targeted to local cells or transported to cells in distant tissues via biological fluids. After binding to recipient cells, EVs may remain stably associated with the plasma membrane, dissociate, directly fuse with the membrane, or be internalized through endocytic pathways [32]. EVs provide the advantages of a stable delivery system well tolerated in biological fluids, the ability to home to target cells or tissues, and are thought to possess negligible immunogenicity *in vivo* [33]. The importance of the EV delivery system was demonstrated where intact, but not lysed, EVs enhanced myocardial viability in a myocardial ischemia/reperfusion mouse injury model [34]. Some studies have demonstrated immunosuppressive behaviour which may be owing to soluble or surface expressed HLA-G, thoroughly reviewed by Rebmann et al. [35].

**Figure 1:**
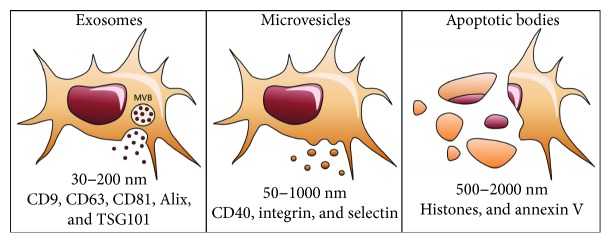
Types of extracellular vesicles and their identifying characteristics. Exosomes, with diameters ranging from 30 to 200 nm, are formed by the inward budding of multivesicular bodies (MVBs), which then fuse with the plasma membrane to be released into the extracellular environment. Exosomes are classified by tetraspanins CD9, CD63, and CD81 and the proteins Alix and TSG101 involved in MVB biogenesis. Microvesicles, also referred to as ectosomes, are larger with diameters from 50 to 1000 nm and bud directly from the plasma membrane. Microvesicles encompass identifying markers CD40, integrin, and selectin. Apoptotic bodies range from 500 to 2000 nm and encompass fragments of dead or dying cells. These are characterized by the presence of histones and annexin V.

EVs is an umbrella term for different types of vesicles secreted by MSCs: exosomes, microvesicles (also referred to as ectosomes), and apoptotic bodies. Explorations of the therapeutic value of EVs are currently focused primarily on exosomes and microvesicles. Each type of vesicle is characterized by its origin, size, and unique identifying markers (Figure 1). The term EVs has been recommended as an inclusive term by the International Society for Extracellular Vesicles (ISEV) as the commonly used methods for isolation of each individual type of EV are not able to exclusively sort one from the other [36].

A majority of research in EVs has focused on the exosome-rich fraction. Exosomes have been described as a relatively homogeneous population in terms of size and are the best characterized among all EVs [37]. The first studies by Timmers et al. [38] provided evidence that MSC-derived CM could provide therapeutic benefits without the use of cells. Different fractionations of conditioned medium were injected into a mouse model of cardiac ischemia and reperfusion injury where it was determined that CM-containing products greater than 1000 kDa were effective in cardioprotection and reduction of infarct size. This size range suggests the involvement of exosomes, which were isolated and tested successfully in a follow-up study [39]. A number of studies have compared such exosome-rich fractions to CM and described comparable results, further indicating that exosomes may be responsible for the therapeutic effects of MSC-derived CM [18, 30, 31]. More recently, exosomes have been compared to microvesicles with variable results. In a model of acute kidney injury, only the exosome-enriched fraction induced an improvement of renal function and morphology [40] while the best formulation to reduce radiation damage to bone marrow stem cells included both types of EVs [41]. Regardless, the beneficial properties of EVs have been attributed to not only their stable delivery of cytokines and growth factors, but also their enclosed RNAs, which play a large role in regulating gene expression to control cell function [30, 31, 42].

### 2.3. Coding and Noncoding RNAs

MSCs secrete protein-coding messenger RNAs (mRNAs) and noncoding RNAs such as microRNAs (miRNAs), long noncoding RNAs (lncRNAs), and circular RNAs (circRNAs) via their extracellular vesicles (EVs). Such components are potentially capable of eliciting changes in function via protein translation or the alteration of gene expression in recipient cells. Recent developments in RNA sequencing and RT-qPCR techniques have enabled the detection of RNAs even in low amounts [42]. Additionally, evidence that mRNAs residing in EVs can be transported into a recipient cell and then translated to contribute to protein expression has had a large impact on the field. For example, kidney tubular cells lacking IL-10 expression exposed to MSC-derived EVs acquired IL-10 mRNA and translated it to the corresponding protein [43].

miRNAs are small noncoding, highly conserved, single-stranded RNAs with function in RNA silencing but are also capable of regulating gene expression through posttranscriptional modifications. miRNAs typically degrade more quickly than do mRNAs. However, they are able to become more stable by associating with RNA-binding proteins (RBPs) or high- and low-density lipoproteins or through EV encapsulation [42]. miRNAs have been shown to be associated with a wide range of biological processes including cell apoptosis, stem cell differentiation, cardiac and skeletal muscle development, hematopoiesis, neurogenesis, insulin secretion, and immune response [44–47]. With such an importance in physiology, miRNA dysfunction can be correlated to disease [48], and consequently it is intensely studied as a diagnostic and prognostic biomarker.

LncRNAs and circRNAs are two more subsets of small RNAs enclosed within EVs. LncRNAs are involved in cellular processes such as chromatic organization, gene transcription, mRNA turnover, protein translation, and the assembly of macromolecular complexes [42]. They have been identified in EVs with differing expression patterns to their parent cells and present specific motifs that appear to complement those of certain miRNAs [42]. Thus, it has been proposed that lncRNAs may capture miRNA subsets and target them into EVs. circRNAs are highly abundant with a long half-life due to their lack of free ends which prevent degradation by exonucleases. Such circRNAs may enable critical transcriptional and posttranscriptional modifications and control miRNA function as regulators of mRNA stability and/or translation [42].

## 3. Bioprocess Development for Secretome-Derived Products

To properly characterize and assess the therapeutic potential of the MSC secretome and the associated EVs, there is a requirement for the standardization of protocols for the expansion of MSC cultures, collection of the secretome, and isolation of defined components. To meet clinical needs for MSC secretome-derived products, isolated MSC populations need to be expanded *in vitro* using defined culture conditions that are reproducible, scalable, and well-controlled to limit heterogeneity and enhance predictability in the composition and function of secretome-derived products. Methods to expand populations of these cells have been developed [49], but have not taken into account the effects on the secretome—now an area of growing importance, particularly in relation to therapeutic efficacy.

**Figure 2:**
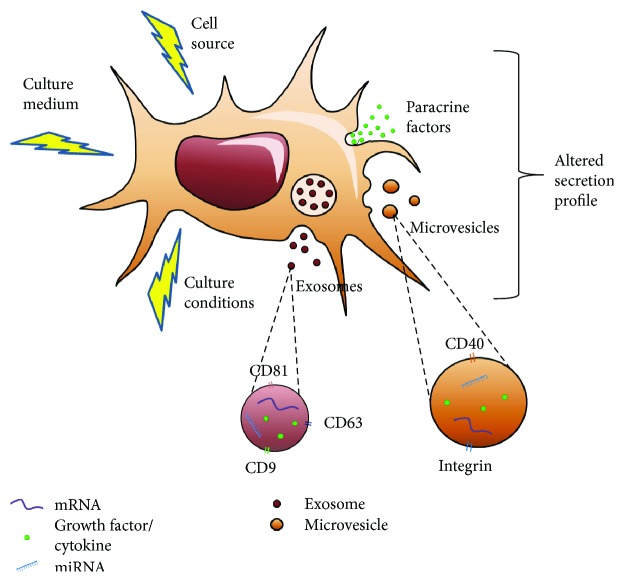
The secretion profile of MSCs may be altered by several factors including culture medium, cell source (i.e., bone marrow and adipose) and culture conditions (i.e., 3D cultures, hypoxia, and mechanical stimuli). The therapeutic portion of the secretion profile includes the amount and composition of paracrine factors and EVs (microvesicles and exosomes). The composition within EVs is also altered which includes RNAs such as mRNAs and miRNAs, growth factors, and cytokines.

Key factors in the development of a cell-based production system include the medium in which the cells are grown, cell source, and culture conditions (Figure 2). Also pertinent are the timing and method of secretome collection, as the secretome is highly dynamic [16]. It is also important to evaluate and develop protocols for the storage, transport, and delivery of secretome-derived products to enable researchers to properly compare and reproduce studies and further the development of therapies and drugs utilizing the MSC secretome. No reliable assay currently exists to test EV membrane integrity, which may impact the therapeutic benefit of the EVs and/or the level of reproducibility after administration [50]. There is also no standard list of biomolecules or RNAs to be quantified, which has resulted in a range of studies that selected their own molecule(s) of interest while disregarding others. Furthermore, there is a need to look at active molecules that may be oncogenic, as the MSC secretome contains proteins and RNAs capable of altering the genome of recipient cells [50]. Despite MSCs having demonstrated therapeutic effects in the treatment of various cancers, there is evidence that certain MSC phenotypes may promote tumor progression and metastasis [51, 52]. The specific mechanisms for crosstalk between MSCs and cancer cells is currently poorly understood, and thus, the oncogenic potential of MSCs and the MSC secretome remains controversial [51, 52].

### 3.1. Culture Medium

A well-defined culture medium is critical to translating MSC secretome-derived products to the clinic. For characterization and analysis of the secretome, a thorough understanding of what is already contained within the medium is required. The majority of studies that culture MSCs report utilizing fetal bovine serum (FBS) in the medium due to the relatively low level of antibodies and high amounts of growth factors that it contains [53, 54]. However, FBS presents high variability in composition depending on where, when, and how it was collected and can also be contaminated with animal-derived infectious agents [55]. Furthermore, when human MSCs are cultured in a medium containing animal proteins, the proteins are retained within the cells and may elicit an immunologic response when the cells or cell products are transplanted [53, 56]. Serum also contains its own exosomes, which must first be removed in exosome and EV-based studies to prevent co-isolation with those derived from the MSCs [57].

Alternatives to FBS include human platelet lysate (HPL) supplementation in media and a variety of chemically defined serum-free media (SFM). Compared to FBS, HPL reduces immunological reactions and enhances the proliferation of MSCs [57]. HPL has a high fibrinogen content that promotes the formation of fibrin gels in calcium-containing media, although this effect can be alleviated by utilizing a recently developed fibrinogen depletion method [57]. Although HPL may represent a cost-effective alternative to FBS for MSC expansion, it has been reported that HPL-expanded MSCs exhibited highly compromised immunosuppressive properties [58]; thus, it is important to fully analyze its effects on the therapeutic properties of MSCs.

A wide variety of chemically defined SFM have been developed for the expansion of human MSCs (hMSCs) that hold more promise, albeit at a higher price tag. Compared to MSCs expanded in serum-containing medium, the defined nature of SFM limits the heterogeneity between batches of cells and enhances MSC proliferation while generating smaller-diameter cells with stable surface marker expression [58, 60]. The difference in therapeutic benefits of secretome-derived products from cells grown in serum-free formulations compared to FBS still needs to be studied. Commercially available serum-free media that have been shown to successfully expand hMSC cultures include StemPro MSC SFM (Invitrogen), MesenCult-SF/XF (Stemcell Technologies), and Becton Dickinson Mosaic hMSC SFM [53, 59, 60]. Of the commercially available SFMs, StemPro MSC SFM is the only FDA-approved serum-free formulation [61]. Additionally, many research labs have developed their own xeno- or serum-free media. PPRF-msc6 is a serum-free medium developed by our laboratory group with a published component list to encourage further research and standardization [62].

### 3.2. Cell Source

MSCs are very broadly defined. For this reason, populations of cells which adhere to this definition can still vary from one another. This variability may be evident when comparing MSC populations sourced from different individuals or even from different tissues within an individual. MSCs also vary in function depending on the tissue from which they are isolated within the body, displaying distinct secretome profiles specific to their native tissue. Further, MSCs can be genetically engineered to enhance the therapeutic benefit of their derived products, as detailed by Hodgkinson et al. [63]. It will be important to match donor characteristics and tissue source to secretome functionality in specific disease models.

#### 3.2.1. Donor-to-Donor Variability

It is well known that inherent variability exists between MSCs derived from different donors/patients, related to factors such as the age and health of the individual [64]. For example, Heathman et al. [65] showed substantial differences in metabolite consumption and production, growth characteristics, and immunoregulation abilities *in vivo* between five different bone marrow-derived MSC lines. Similarly, Paladino et al. [66] described unique behaviour of Wharton jelly-derived MSCs derived from different individuals, exhibiting differing cytokine profiles and immunomodulatory capacities. Phenotype, donor age, and gender have all been found to be contributing factors in the function of MSCs [67].

The metabolic state of the individual is another large factor found to influence MSCs and their secretome-derived products, with exosomes in particular being associated with metabolic organ crosstalk [68]. Between adipose-derived MSCs from lean and obese patients, distinct expression patterns of stem cell markers and varied lncRNA expression levels within exosomes were found [69]. Obesity was further found to reduce the proangiogenic potential of adipose MSC-derived EVs, showing reduced amounts of VEGF, MMP-2, and miR-126 within the EV cargo [70]. Interestingly, it has been found that MSCs derived from type 2 diabetes mellitus (T2DM) patients display no difference in cell surface marker phenotype, morphology, or multilineage potential compared to healthy individuals, but have decreased potency, oxidative stress-dependent dysfunctions, and a dysfunctional secretome composition that enhances proangiogenic function [71].

In comparing MSCs derived from healthy individuals to those from diseased states, it is not surprising that those from diseased states exhibit reduced function. For example, compared to a healthy control, CM obtained from MSC cultures using cells derived from multiple sclerosis (MS) patients eliminated the neuroprotective effect of MSC-CM when used in a model of progressive MS [72]. In some cases, such as in MSCs derived from T1DM patients, the MSCs show no differences in terms of morphology, immune-suppressive activity, and migration capacity, but had gene expression differences that could have impacted their *in vivo* function [71]. The mechanisms by which donor characteristics such as age and gender, metabolic state, and disease alter MSC function and their corresponding secretome are currently not well understood. Further understanding the impact of these factors will be crucial to the development and application of secretome-derived products.

#### 3.2.2. Tissue Source

Proteomic comparisons of the secretomes of MSCs derived from different tissue sources have revealed differing secretome profiles. Between bone marrow, adipose tissue, and dental pulp-derived MSCs, only 124 of 1533 identified proteins were common across all three sources [73]. These commonly secreted proteins are factors with functions linked to MSC-related biological effects. A different comparative analysis among bone marrow, adipose tissue, and umbilical cord perivascular cells revealed differing secretome profiles of neuroregenerative factors [1]. One study showed Wharton's jelly-derived MSCs secrete greater amounts of cytokines, proinflammatory proteins, and growth factors, while those derived from adipose tissue have an enhanced angiogenic profile and secrete greater amounts of extracellular matrix (ECM) proteins and metalloproteinases [74]. The enhanced angiogenic profile of adipose-derived MSCs was also confirmed by Hsiao et al. [2]. Within the literature, the profiles secreted by different source-derived MSCs are relatively consistent, with embryonic or umbilical-derived stem cells showing enhanced proliferative and developmental molecules, and those from adult sources, such as bone marrow and adipose tissue, secreting higher amounts of ECM maintenance-related proteins [15].

In terms of EVs, fewer studies have compared profiles of different MSC sources to date. Bone marrow- and adipose tissue-derived MSCs secrete exosomes with highly similar RNA expression profiles, but with distinctive enrichments in specific tRNAs [75]. Compared to bone marrow, umbilical cord, and chorion-derived stem cells, exosomes secreted by menstrual-derived MSCs were shown to enhance neurite outgrowth response, relevant to recovery from neurodegenerative disorders such as Parkinson's disease [76]. Cell source is clearly an important aspect of process development which needs to be tailored towards specific therapeutic targets. Further studies need to be done to correlate the impact of different cell sources towards the therapeutic benefit for various disease models.

### 3.3. Culture Conditions

The characteristics of MSCs are impacted by environmental parameters including temperature, pH, cell density at which they are seeded, oxygen level, and any mechanical, electromagnetic, or biochemical stimuli to which they are exposed. Culture conditions may function as a regulator to generate a certain MSC population with characteristics suitable for a particular application. Consequently, it is important to match culture conditions to the specific intended application. Similarly, culture conditions also impact the composition and bioactivity of the MSC secretome in culture. Therefore, steps need to be taken to ensure that a particular set of culture conditions results in secretome-derived products valuable for a specific application.

One way to alter the culture environment is to change the platform on which the cells are grown. Tissue culture flasks (T-flasks) provide a simple means of cell population expansion and are commonly used in small-scale research studies. In T-flasks, MSCs adhere to the surface and grow under static conditions as a 2D monolayer. However, when considering scalability towards clinical applications, the large number of T-flasks needed can lead to flask-to-flask variability, increases the chance of contamination, and can be labour-intensive [53]. Another common platform for expanding large populations of cells is a stirred suspension bioreactor, where the cells are grown in suspension in the presence of mechanical agitation. MSCs are traditionally grown in suspension bioreactors as an adherent monolayer by adding small beads called microcarriers on which the cells can attach and grow [56]. Suspension bioreactors offer a higher level of homogeneity and process control which serve to reduce both batch-to-batch and within-batch variability of cell cultures. Furthermore, stirred suspension bioreactors are highly scalable and several variables such as dissolved oxygen, pH, and temperature can be computer-controlled to provide a high level of process control and thus more uniform batches of products. The use of bioreactor technology has been thoroughly reviewed by Schnitzler et al. [56].

**Figure 3:**
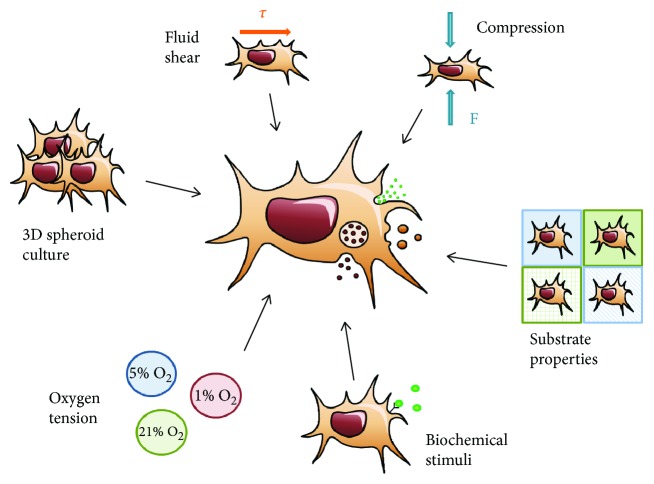
Alterations to MSC cultures reported to have an influence on the MSC secretome profile. 3D spheroid culture (i.e., forced cell-cell interactions), fluid shear, compression forces, the properties of the cells' residing substrate (i.e., stiffness and topography), biochemical stimuli (i.e., exposure to inflammatory factors), and the amount of oxygen cells are exposed to influence the amount and types of biomolecules secreted by MSCs.

A wide variety of platforms and methods are available to grow MSCs. The differing effects of these platforms and methods should be realized in early stages of development to ensure scalable and effective clinical translation. It is important to further consider the implications of differing culture conditions within the chosen culture platform to effectively optimize product development. The MSC secretome can be tailored through altering culture conditions such as forced cell-cell interactions, oxygen level, and exposure to mechanical forces or biochemical factors (Figure 3), as described below [24, 77]. Advances in understanding the effect of differing culture conditions on the MSC secretome and/or its enclosed EVs are summarized in Table 2.

#### 3.3.1. Three-Dimensional Spheroid Culture

MSCs can be induced to grow as three-dimensional (3D) aggregates (spheroids) where the cells attach to each other instead of a surface. The most common method to create spheroids is the hanging drop method, in which small droplets of cell suspension are placed on a static tissue culture flask and cultured upside down over a bath of buffer solution such as phosphate-buffered saline (PBS). Spheroids have also been created spontaneously in low attachment plates, in microwell-based systems such as AggreWells, and in suspension bioreactors by inoculating the cells at a high density [77–79]. Compared to traditional 2D adherent monolayer cultures, growth as 3D spheroids is considered more physiologically relevant [80]. MSCs within 3D aggregates have been shown to exhibit enhanced anti-inflammatory, angiogenic, and tissue reparative/regenerative properties [80]. The mechano-physical properties in MSC spheroids are drastically different, with cytoskeletal reorganization and changes in cell morphology which create relatively smaller cells with a spherical shape. MSCs grown within spheroids also have significant differences in gene expression and enhanced stem cell properties (i.e., stemness) including improved multidifferentiation potential [81].

Similarly, it has been demonstrated that CM derived from spheroid MSC cultures is more effective than MSCs grown as a monolayer in suppressing an inflammatory response in stimulated macrophages in coculture and in a mouse model [28, 82]. This effect has been attributed to significantly higher expression levels of anti-inflammatory factors TSG-6, STC-1, and CXCR4 and increased secretion of PGE2 as the principal mediator of inflammation. CM from spheroid MSCs inhibited macrophages from secreting proinflammatory cytokines TNF-*α*, CXCL2, IL-12p40, and IL-23 and increased their secretion of anti-inflammatory cytokines IL-10 and IL-2R*α*. Another study revealed a significant decrease in the chemotactic index of CD14^+^ cells when incubated with 3D MSC spheroid-derived microvesicles compared to 2D MSC-derived microvesicles. This suggests that MSC culture mode can impact the immunomodulatory characteristics of the resulting microvesicle population [83].

MSCs cultured as 3D spheroids also exhibited an increased level of certain proteins and cytokines with more than purely immunomodulatory effects, such as antioncogenic proteins IL-24, TNF-*α*, CD82, vasculogenesis and angiogenesis promoter VEGF, and proteins involved in cell differentiation and survival such as TGF-*β*3 [82, 83]. Furthermore, MSCs grown in 3D spheroids cocultured with osteoarthritic chondrocytes exhibited higher potential for cartilage repair compared to those grown as a 2D monolayer [84]. Although there is limited research on the effect of spheroid culture on many aspects of the secretome, one would expect the increase in cell-cell interaction, altered cell morphology, and the potentially hypoxic nature of the spheroid's internal microenvironment to have a large effect on the composition of the resulting MSC secretome.

#### 3.3.2. Hypoxia/Anoxia

The majority of *in vitro* cultures are exposed to headspace oxygen levels of 21%. Since MSCs are typically not exposed to such high levels of oxygen in their native environment, this oxygen level can be considered to be hyperoxic, a culture condition that has been reported to contribute to oxidative stress and genetic instability resulting in DNA damage and reduced lifespan [85]. Anoxia on the other hand, defined as a headspace oxygen level of <1%, is used to simulate ischemic injury conditions *in vitro*. Whereas MSC survival rates decrease with increased exposure times to anoxia, the lack of oxygen can upregulate the release of chemotactic and angiogenic mediators of crucial importance for tissue regeneration and repair applications [86]. While not necessarily directly reflective of the oxygen levels to which the cells are exposed, conditioning of MSCs at headspace oxygen concentrations of 1–5%, often referred to as hypoxic culture conditions, is employed as a less extreme method than anoxia to induce the activation of survival pathways and the secretion of products that may adapt the cells to their stressed environment [24]. This has resulted in populations that exhibit increased growth kinetics, greater differentiation capacity, and therapeutically desirable characteristics via the activation of hypoxia-inducible factor- (HIF-) 1*α* [87].

Hypoxia has been reported to enhance both the secretion profile of MSCs and the quantity of exosomes released [88–90]. An overexpression of miRNAs involved in inflammatory, proliferative, and differentiative phases has been observed, including miR-223, -146b, -126, -199a, -11, -22, -24, and -210 [89, 91]. This is consistent with an upregulation of factors involved in cellular proliferation, differentiation, survival, angiogenesis, immunomodulation, and/or neuroregulation including VEGF, GM-CSF, IGF-1, IL-6, EGF, FGF, PDGF, and GCSF [88, 90, 92, 93]. Furthermore, *in vivo* studies have demonstrated enhanced muscle regeneration and an elevated protective effect on endotoxin-induced acute lung injury with injection of hypoxia-preconditioned MSC-derived EVs [89, 94].

There are some discrepancies between studies in terms of the upregulation or downregulation of factors resulting from differing oxygen concentrations, exposure times, cell source, or culture environment. Paquet et al. [86] reported that CM from anoxic conditions (0.1% O_2_ headspace) had enhanced chemotactic and proangiogenic properties, along with a reduced inflammatory mediator content, while it showed no substantial differences between hypoxic (5% O_2_ headspace) and normoxic (21% O_2_ headspace) conditions, contrary to other studies. TGF-*β*1 was reported to be upregulated at a headspace concentration of 1% O_2_ [93] but downregulated at 5% O_2_ [92]. Hung et al. [93] also showed upregulated osteogenic and adipogenic factors, but a decrease in chondrogenic factors, contrary to several studies that demonstrated enhanced chondrogenesis in hypoxia-induced MSCs [95–97]. Li et al. [94] demonstrated the importance of exposure time in a study where they exposed MSCs to a hypoxic environment for 30, 60, or 90 minutes with differing resultant secretome profiles.

The MSC secretome is highly dynamic, and there is a clear need for accurate reporting in oxygen tension studies. Although studies report the oxygen concentration in the air, the dissolved oxygen levels to which cells are exposed in culture may differ depending on parameters such as depth of medium, cell density and oxygen consumption rate. The issue of exposure time is also often overlooked. There are differences between cells exposed to short-term hypoxic preconditioning and those that have undergone long-term expansion in low oxygen environments. Cells exposed to hypoxia from passage 0 to passage 2 were reported to be able to proliferate faster compared to those cultured in normoxia, and displayed enhanced expression of genes involved in ECM assembly, neural and muscle development, and epithelial development [98]. With MSCs exhibiting altered growth characteristics and gene expression, it is likely that the secretome profile would also be altered in such circumstances. It is clear that hypoxic conditions heavily affect the therapeutic properties of MSCs, but standardization of hypoxia-related protocols is needed to properly compare and reproduce results.

#### 3.3.3. Mechanical Stimuli

MSCs have been shown to be highly mechanosensitive [99]. Cell behaviours such as proliferation and differentiation, as well as their secretome profile, have been shown to be strongly influenced by mechanical stimuli such as fluid shear stress and compression [24, 100]. MSCs transfer mechanical stimuli from their surrounding microenvironment into biochemical signals via mechanotransduction [100]. While the majority of publications in this area have focused on mechanical stimulation as a means of impacting cellular differentiation [99, 101], it is increasingly evident that the paracrine factors generated by the cells are highly influenced by their mechanical environment [16, 100, 102, 103]. For example, culturing MSCs on microcarriers within stirred suspension bioreactors, where cells are exposed to fluid shear forces, was found to enhance the neuroregulatory profile of the secretome, including a number of CNS regulators only detected in the CM of bioreactor-cultured MSCs and not in the CM of cells grown in static tissue culture plates [16]. Classic trophic factors BDNF, VEGF, and IGF-1 were also upregulated in dynamic bioreactor culture [16]. MSC constructs exposed to physiological compression had an enhanced angiogenic profile within the CM, which was also enriched with the soluble regulators MMP-2, TGF-*β*1, and bFGF [102]. Further, the exposure of MSCs to multiaxial shear and compression enhanced their chondrogenic profile similarly, but distinctly, to that caused by TGF-*β*1 stimulation [100]. Nitric oxide (NO) production was significantly higher in loaded groups, with NO playing an effective role in MSC immunomodulation through the suppression of T cell response [100].

MSCs also respond to the mechanical properties of their substrate (i.e., the surface on which they are attached) including rigidity [99]. Matrix stiffness can guide differentiation of cells towards specific lineages [99], and as such, changes to cell state alter the secretome [100]. VEGF, angiogenin, and IGF are upregulated with increasing elastic moduli, whereas EGF, IL-6, and IL-8 levels are not responsive to the change of stiffness [103]. It is apparent that mechanical stimulation plays a significant role in determining the composition and function of the secretome, and further research should explore ways to take advantage of dynamic cultures and the mechanical properties of cell culture substrates.

#### 3.3.4. Electromagnetic Stimuli

Exposure of MSCs to electromagnetic fields (EMFs) is another strategy to influence cell behaviour as cells communicate with each other through sending and receiving electromagnetic signals [104]. In collagen-rich tissues, such as bone and cartilage, small, endogenous electric fields are produced during applied mechanical stresses [105]. Therefore, most research involving MSCs in this area to date has focused on osteogenic and chondrogenic differentiation through EMF stimulation [106]. For example, exposure of Wharton jelly-derived MSCs to EMF (1.8 mT, 75 Hz, 8 h/day for 21 days) increased cell division and cell densities, induced early chondrogenic differentiation, and increased collagen II expression levels [107]. Similarly, though dependent on intensity, time of exposure, and frequency of EMF, EMF exposure has been used to enhance osteogenic and neural differentiation [108–110], impact cell metabolism and structure [111], and increase cell viability [112].

Very little work has focused on the effect of EMF on MSC secretions. Marędziak et al. [113] reported that under static magnetic field (0.5 T), adipose-derived MSCs secreted a considerably higher number of microvesicles compared to a control with no magnetic stimulation. In addition, microvesicles collected from the magnetically stimulated MSCs contained higher amounts of BMP-2, VEGF, and p53 and lower amounts of TNF-*α*. Thus, controlling EMF exposure, along with mechanical, electrical, and biochemical stimuli, may provide a significant opportunity to enhance and optimize bioprocesses for MSC secretome-derived products.

#### 3.3.5. Biochemical Stimuli

MSCs are also highly influenced by direct biochemical signals elicited by various biomolecules. Much of the research involving the MSC secretome in terms of biochemical signalling is based around the fact that stimulated MSCs release molecules and EVs to counteract such biological signals and, thus, produce higher amounts of supportive proteins, miRNA, lipids, and other metabolites [114]. One way to induce a proinflammatory stressed phenotype is to precondition MSCs with lipopolysaccharide (LPS) [115, 116]. In traditional 2D monolayer cultures, compared to a control of MSCs cultured without LPS, LPS preconditioning induced MSCs to release proinflammatory cytokines IL-1*β*, IL-6, IL-8, IL-12, IFNs, and TNF-*α*, which, in turn, lowered the secretion of proinflammatory cytokines from other cell types [115, 116]. LPS-preconditioned MSC-derived exosomes were injected in a cutaneous wound model in streptozotocin-induced diabetic rats and were able to upregulate the expression of anti-inflammatory cytokines and promote M2 macrophage activation [115]. In partially hepatectomized mice, intravenously administered CM from LPS-preconditioned MSCs lowered the secretion of proinflammatory cytokines IL-6 and TNF-*α* and enhanced liver regeneration [116].

MSCs also produce immunomodulatory and regenerative factors in response to inflammatory stimuli such as interferon gamma (IFN-*γ*) and TNF-*α*. IFN-*γ* exposure has been shown to enhance immunosuppressive properties of MSCs by enhancing or inducing MSC inhibitory factors, downregulating T cell activation, enhancing T cell negative signalling, altering T cells from a proinflammatory to an anti-inflammatory phenotype, interacting with antigen-presenting cells, and increasing or inducing regulatory cells [117]. It was, however, shown that IFN-*γ* alone did not induce immunosuppression, and only when combined with either TNF-*α*, IL-1*α*, or IL-1*β* did immunosuppression by MSCs occur to inhibit the proliferation of T cells [118]. With any one of these combinations, MSCs produced several chemokines including CXCL-9 and CXCL-10 in large amounts [118]. Also, for MSCs treated with both IFN-*γ* and TNF-*α*, the secretion levels of IL-6, HGF, VEGF, and TGF-*β* were significantly increased, and the secretion levels with the combination of the two were significantly higher than that of either IFN-*γ* or TNF-*α* on their own [119]. From a different perspective, TNF-*α*-preconditioned exosomes promoted the proliferation and osteogenic differentiation of human primary osteoblastic cells [120].

Hydrogen peroxide (H_2_O_2_) can be used to induce an ischemia-mimicking microenvironment. Though high concentrations of H_2_O_2_ can induce cell death or damage, low concentrations may trigger processes that provide protective effects against stressful conditions. Stimulation with H_2_O_2_ has been shown to increase the expression of proangiogenic proteins such as VEGF and HGF [121], as well as the secretion of IL-6 [122]. Bai et al. demonstrated that MSCs stimulated with H_2_O_2_ enhanced their angiogenic effect on HUVECs and increased skin flap survival in an *in vivo* model [121].

Transforming growth factor- (TGF-) *β*1 has links to cellular proliferation, differentiation, and both anti- and proinflammatory effects. TGF-*β*1 exposure modulates the MSC secretome by altering the secretion of cytokines and chemokines (listed in Table 2) involved in immunosuppression, allergic response, and bone resorption [123]. Such studies provide evidence that the secretome can be modulated by biochemical factors, but a significant amount of research still needs be done to evaluate how exposure time and dose of biochemical stimulation impact the resulting paracrine factor profile for MSCs.

### 3.4. Isolation

The isolation of MSC secretome-derived products is an important consideration in bioprocess development because of the highly dynamic nature of the secretome. The type and amount of products secreted depend on the culture period, or culture growth phase, from which the secretome is isolated. The method of isolation of specific products, primarily of EVs within the medium, also needs to be considered. Many challenges exist with current methods for EV isolation, as recently reviewed by Li et al. [124] and Gholizadeh et al. [125]. Ultracentrifugation, while able to deal with relatively large volumes, and currently considered to be the gold standard for separating these structures, only sees recovery rates of up to 25% and is a long cumbersome process [126]. There is also evidence that the high forces involved (typically 100,000*g*) can affect the bioactivity of the EVs themselves [127]. Size exclusion and antibody-based capture mechanisms are limited by a low throughput and the need for a final concentration step [128]. Furthermore, the use of antibody-based mechanisms is limited in many applications due to high cost. Microfluidics approaches enable simultaneous isolation and characterization of EVs, but EV recovery from such devices remains challenging [125]. More recently, anion-exchange chromatography has been used to isolate EVs in a single step with high purity, which exploits the negative charge of EVs [129]. There is a great need for EV isolation technologies that enable high throughput, maintain EV integrity and bioactivity, and are not cost prohibitive for clinical applications. It will be important to take advantage of the intrinsic properties of EVs to develop cost-effective isolation methods going forward.

### 3.5. Storage

For the storage and transport of MSC secretome-derived products, it is important to consider the effects of freeze-thaw, stability at various temperatures, and the effects of freeze-drying components. CM can reportedly be stored at −20°C for several months without experiencing functional deterioration [130]. For EVs, it has been shown that freeze-thaw does not affect the size of exosomes or impair the membrane integrity, but there is 60% reduction in size after 2 days at a physiological temperature of 37°C as well as a reduction after 3–4 days at 4°C [131]. When frozen at −20°C, exosomes are stable for periods as long as 6 months without a loss in their biochemical activity [9]. However, a study by Zhou et al. [132] demonstrated that protease inhibitors were essential for proper preservation, and freezing urinary exosomes at −20°C resulted in major losses, while freezing at −80°C enabled almost complete recovery after up to 7 months of storage. Further, extensive vortexing (i.e., 90 seconds) enabled maximum recovery of thawed exosome samples. These studies demonstrate that exosomes can undergo long-term storage with high recovery without a loss in bioactivity. Compared to cells, which exhibit impaired therapeutic properties as a result of freeze-thaw and require the use of preservatives for proper cryostorage, secretome-derived products are more amenable to storage, a key consideration from a translational perspective [53].

### 3.6. Delivery

*In vivo* delivery of secretome-derived products offers similar limitations to cell therapies. The majority of studies utilize simple intravenous injection of culture media or EVs in PBS into the site of injury. Despite the ability of exosomes to home to target tissues, there is limited retention in these areas, and repeated injections need to be performed for effective treatment [133]. One publication outlined the use of a photoinduced imine-cross-linking hydrogel which could be embedded with exosomes and used as a hydrogel tissue patch [133]. Cells were able to migrate into the hydrogel and internalize the encapsulated exosomes. Gangadaran et al. [134] also demonstrated enhanced EV retention within an injury site when mixed and injected with a Matrigel scaffold. Such a system prevents migration of cell-derived products, such as exosomes, from the target site and enables better integration for tissue repair. Depending on the application of such cell-derived therapeutics, hydrogel systems may enable more effective treatments via the slow release of products to prevent rapid site clearance or premature degradation.

MSC-derived exosomes have also been successfully delivered via integration into tissue-engineered bone [135] and within a fibrin surgical mesh [136]. The exosomes were added to enhance healing and to encourage migration of cells into the scaffolds. While the primary goals of these studies were to improve the outcomes of such constructs, the success of EV integration and retention offers an additional perspective for the use of cell-free therapeutics.

## 4. Conclusions

With recent reports attributing the main therapeutic benefits of MSCs to their paracrine effects, the secretome and its constituent exosomes may represent a novel and safer approach for the treatment of medical conditions compared to direct cell-based therapies. The fact that the secretome and its components are nonliving will facilitate the development of therapeutic strategies that can ensure higher sterility, lower immune response, and the ease of transport and storage. Understanding how cell source, media, and culture conditions interact to determine the quantity, quality, and types of biomolecules secreted is essential to the development of bioprocesses aimed at scaling up the production of secretome-derived products. Furthermore, standardization of protocols for isolation, characterization, storage, and delivery are needed to properly compare studies and ensure effective quality control.

## Figures and Tables

**Table 1:** Biological effect of MSC secretome-derived products on disease models.

MSC source	Paracrine factors	Biological effect	Ref.
Skin wounds and radiation			
Human adipose tissue	Supernatant of cell lysate	(i) Faster wound closure when applied topically on cutaneous wound (ii) Upregulation of dermal fibroblast proliferation, migration, and ECM production	[25]
Human adipose tissue	Hypoxic conditioned medium	(i) Protected epithelial, endothelial, and myoepithelial cells from radiation damage and tissue remodelling	[92]
Adipose tissue	Exosomes	(i) Stimulated fibroblast migration, proliferation, and collagen synthesis (ii) Recruited to soft tissue wound in mouse skin incision model and accelerated cutaneous wound healing	[137]
Human and murine bone marrow	Exosomes and microvesicles	(i) Mitigated radiation injury to marrow stem cells (ii) Restoration of marrow stem cell engraftment and partial recovery of peripheral blood counts postirradiation	[41]
Human amniotic epithelial cells	Exosomes	(i) Promoted migration and proliferation of fibroblasts (ii) Deposition of ECM partly abolished (iii) In rat model, improved skin wound healing with well-organized collagen fibers	[138]
Human umbilical cord blood	Exosomes	(i) Promoted cell migration and collagen synthesis of human dermal fibroblasts (ii) Increased expressions of collagen I and elastin 3 days posttreatment on human skin	[139]

Bone and cartilage			
Human fetal MSCs	Conditioned medium	(i) Increased expression of ALP and osteogenic marker genes and increased calcium deposits in rat BM-MSCs (ii) Improved bone consolidation in a rat osteogenesis model	[26]
Human synovial membrane	Exosomes	(i) Enhance proliferation and antiapoptotic abilities of bone marrow-derived stromal cells (ii) Prevented GC-induced trabecular bone loss, bone marrow necrosis, and fatty cells accumulation in rat model	[140]
Human embryo	Exosomes	(i) Enhanced gross appearance and histological scores of osteochondral defects in adult rats with complete restoration of cartilage and subchondral bone	[141]
Human bone marrow	Exosomes compared to exosome-free conditioned medium	(i) Exosomes, but not exosome-free conditioned medium, rescued retardation of fracture healing in CD9^−/−^ mice	[31]
Human iPS-MSCs	Exosomes	(i) In a rat osteonecrosis model, exosomes prevented bone loss and increased microvessel density (ii) Enhanced proliferation, migration, and tube-forming capacities of endothelial cells in vitro	[142]
Human bone marrow	Exosomes, miR-21	(i) Suppressed TNF-*α*-induced nucleus pulposus cell apoptosis	[46]

Kidneys			
SD rat bone marrow	Conditioned media compared to MSCs	(i) In an acute kidney injury model, MSCs and their CM equally ameliorated kidney function deterioration, Kim-1 shedding in urine, renal tissue damage, and tubular cell apoptosis (ii) Both reduced interstitial fibrosis	[27]
Bone marrow	Conditioned medium, MSCs, and microvesicles	(i) Ameliorated induced acute kidney injury in rats with little differences in effectiveness between CM, microvesicles, and MSCs	[143]

Diabetes mellitus			
Murine bone marrow	miR-106b-5p, miR-222-3p	(i) Promoted postinjury *β*-cell proliferation (ii) Improved hyperglycemia in STZ-treated mice	[47]
Human adipose tissue	Conditioned media compared to MSCs	(i) Reversed mechanical, thermal allodynia, and thermal hyperalgesia (ii) Restored correct pro/anti-inflammatory cytokine balance and prevented skin innervation loss (iii) Reestablished Th1/Th2 balance in spleens of STZ-treated mice (iv) Recovered kidney morphology	[144]
Human bone marrow	Extracellular vesicles	(i) Prevented onset of T1DM and experimental autoimmune uveoretinitis in a murine model (ii) Inhibited activation of antigen-presenting cells and suppressed development of Th1 and Th17 cells	[145]

Cardiovascular system			
Human embryonic MSCs	Exosomes	(i) Reduced infarct size in a mouse model of myocardial ischemia/reperfusion injury	[39]
SD rat bone marrow	Exosomes compared to MSCs	(i) Exosomes reduced inflammation, inhibited fibrosis, and improved cardiac function in rat myocardial infarction model (significantly superior to MSCs) (ii) Exosomes stimulated cardiomyocyte H9C2 cell proliferation, inhibited apoptosis, and inhibited fibroblast differentiation to myofibroblast	[18]
SD rat bone marrow overexpressing Akt	Hypoxic conditioned medium	(i) Suppressed hypoxia-induced apoptosis and triggered contraction of adult rat cardiomyocytes (ii) Upregulation of VEGF, FGF-2, HGF, IGF-1, and TB4 in Akt-MSCs	[146]
Human bone marrow	Conditioned medium—products >1000 kDa (100–220 nm)	(i) Cardioprotection in a mouse model of ischemia and reperfusion injury with a 60% reduction in infarct size (ii) Reduced myocardial nuclear oxidative stress (iii) Reduced TGF-*β* signalling and apoptosis (iv) Improved systolic and diastolic cardiac performance	[38]
huES9.E1	Exosomes	(i) Alleviated features of reperfusion injury (ii) Preservation of left ventricular geometry and contractile performance (iii) Increased levels of ATP and NADH and decreased oxidative stress (iv) Reduced local and systemic inflammation (v) Reduced infarct size by 45%	[34]
Murine bone marrow	Exosomes enriched in miR-22 from ischemic preconditioned MSCs	(i) Reduced cardiac fibrosis in a myocardial infarction mouse model (ii) Mobilized to cardiomyocytes where they reduced apoptosis due to ischemia	[91]
Human umbilical cord	Exosomes	(i) Improved cardiac systolic function and reduced cardiac fibrosis after litigation of LAD coronary artery in a rat model (ii) Protected myocardial cells from apoptosis and promoted tube formation	[147]
SD rat bone marrow	Exosomes from GATA-4-overexpressing MSCs, miR-19a	(i) Restored cardiac contractile function and reduced infarct size following ligation of coronary artery in rat heart (ii) Increased cardiomyocyte survival and preserved mitochondrial membrane potential	[148]
Murine bone marrow	Extracellular vesicles, miR-210	(i) Improved angiogenesis and exerted a therapeutic effect on myocardial infarction in a mouse model (ii) miR-210 necessary for proangiogenic effect	[45]
SD rat bone marrow	Exosomes	(i) Enhanced tube formation of human umbilical vein endothelial cells (ii) Impaired T cell function by inhibiting proliferation in vitro (iii) Reduced infarct size, preserved cardiac systolic and diastolic performance, and enhanced density of new capillaries in a rat myocardial infarction model	[149]
SD rat bone marrow	Exosomes	(i) Reduced H_2_O_2_-induced ROS production and cell apoptosis of rat H9C2 cardiomyocytes	[150]
Human bone marrow	Exosomes from ischemic MSC culture conditions	(i) Induced angiogenesis via NF*κ*B pathway in HUVECs	[88]
Human Wharton jelly	Microvesicles	(i) Improved survival rate and renal function in renal ischemia-reperfusion injury after cardiac death (ii) Decreased number of CD68^+^ macrophages in kidney (iii) Decreased protein levels of *α*-SMA and TGF-*β*1 and increased HGF levels	[151]
Murine bone marrow	Extracellular vesicles	(i) Increased blood reperfusion and formation of new blood vessels in a hindlimb ischemia model	[134]

Cancer			
Human embryonic kidney cell line 293	GE11-positive exosomes containing miR-let-7a	(i) Suppressed tumour growth and development in tumour-bearing mice (ii) Delivered miRNA to EGFR-expressing xenograft breast cancer tissue	[152]

Muscle injury			
Human bone marrow	Conditioned media compared to exosomes	(i) Promoted myogenesis and angiogenesis in vitro (ii) Exosomes promoted muscle regeneration in a mouse muscle injury model	[30]
Human adipose tissue	Extracellular vesicles	(i) Modulated anti-inflammatory effects inducing macrophage polarization (ii) Mitigated inflammatory milieu within injured tissues in CTX injury of mouse TA muscle (iii) Accelerated muscle regeneration process	[89]

Immunomodulatory			
Human umbilical cord blood	Microvesicles	(i) Decreased chemotactic index of CD14^+^ cells (enhanced immunomodulatory effect)	[83]
Human bone marrow	Conditioned medium, PGE2	(i) CM from spheroids inhibited LPS-stimulated macrophages from secreting proinflammatory cytokines and increased their production of anti-inflammatory cytokines	[28]

CNS			
Human bone marrow	Exosomes	(i) Promoted survival of retinal ganglion cells (RGCs) and regeneration of their axons (ii) Partially prevented RGC axonal loss and dysfunction	[153]
Human bone marrow	Exosomes from hypoxic MSCs	(i) Intravitreal exosome treatment in a oxygen-induced retinopathy murine model partially preserved retinal vascular flow in vivo and reduced retina thinning	[154]
Bone marrow	Exosomes	(i) In T2DM rats, stroke treatment 3 days poststroke improved functional outcome and reduced blood brain barrier leakage and haemorrhage (ii) Increased axon and myelin density and oligodendrocyte and oligodendrocyte progenitor cell number (iii) Increased expression of ABCA1 and IGFR1	[155]
Human adipose tissue	Conditioned medium	(i) Protected SH-SY5Y neuron-like cells against H_2_O_2_-induced neurotoxicity (ii) Promoted recovery of normal axonal morphology, electrophysiological features, and cell viability	[156]
SD rat bone marrow	Extracellular vesicles	(i) Promoted functional recovery and nerve regeneration of crush-injured sciatic nerves in rats	[157]
Wistar rat bone marrow	Conditioned medium	(i) Enhanced motor functional recovery, increased spared spinal cord tissue, enhanced GAP-43 expression, and attenuated inflammation after spinal cord injury in a rat model	[158]

Pulmonary			
Bone marrow	Exosomes	(i) Reduced levels of white blood cells and neutrophils to bronchoalveolar lavage fluid in endotoxin-injured mice	[94]
Human bone marrow	Microvesicles	(i) Reduced symptoms of idiopathic pulmonary fibrosis such as reduced collagen deposition and inflammation in mouse fibrosis model	[159]
Human Wharton jelly, bone marrow	Exosomes	(i) Ameliorated alveolar simplification, fibrosis, and pulmonary vascular remodelling in a hyperoxia-exposed mouse model (ii) Suppressed proinflammatory macrophage M1 state and augmented anti-inflammatory M2 state	[160]
SD rat bone marrow	Microvesicles	(i) Alleviated PAH in a rat model by regulating the angiotensin system (ii) Relieved pulmonary artery pressure, pulmonary vessel wall thickness and lumen area, right ventricular hypertrophy, inflammation, and collagen fiber volume	[161]

Liver			
Human umbilical cord	Exosomes	(i) Reduced surface fibrous capsules and alleviated hepatic inflammation and collagen deposition in a mouse model of CCl4-induced liver fibrosis	[162]

**Table 2:** Effects of differing culture conditions on the MSC secretome. The results shown are in comparison to the secretome of control cells cultured as a 2D monolayer in static tissue culture flasks under normoxic (21% O_2_) conditions. The medium listed does not include antibiotics or antimycotics.

MSC source	Culture mode	Medium	Results	Ref.
3D spheroid cultures				
Human femoral heads	3D spheroid culture in spinner flasks and rotating wall vessels	*α*MEM + 15% FBS	(i) Decrease in surface marker expression levels (ii) Decreased cell size (iii) Enhanced osteogenic and adipogenic differentiation (iv) Differing gene expression profile	[77]
Human umbilical cord blood	Spheroids (hanging drop method)	DMEM + 10% FBS, 1% L-glutamine	(i) IL-2R*α*, IL-7, IL-16, MCP-3, TGF-*β*3, and VEGF detected only in spheroid CM (ii) Significant increase in IL-6, MCP-1, LIF, G-CSF, and SDF-1*α* (iii) Decrease in TGF-*β*1 and TGF-*β*2 levels (iv) Decreased chemotactic index of CD14^+^ cells (v) Enhanced capability to promote signal factors secretion	[83]
Human bone marrow	3D spheroids (hanging drop method)	CCM + 17% FBS	(i) More effective in suppressing inflammatory responses in the coculture system with LPS-activated macrophages (ii) Maximally expressed TSG-6 (iii) Expressed high levels of stanniocalcin-1, IL-24, TNF-*α*-related apoptosis inducing ligand, and CD82 (iv) 1/4 of the volume of monolayer cells	[82]
Human bone marrow	3D spheroids (hanging drop method)	CCM + 17% FBS	(i) Inhibited LPS-stimulated macrophages from secreting proinflammatory cytokines TNF-*α*, CXCL2, IL-12p40, and IL-23 (ii) Increased secretion of anti-inflammatory cytokines IL-10 and IL-1r*α*	[28]
Human adipose tissue	3D spheroids in suspension using ultra low attachment plates	*α*MEM + 10% FBS	(i) Enhanced production of VEGF, SDF, and HGF (ii) Lowered expression of proapoptotic markers	[78]

Oxygen tension (hypoxia/anoxia)				
Human adipose tissue	Hypoxia (1% O_2_)	DMEM	(i) Higher HIF-1*α* expression (ii) Increased release of EVs (iii) Induced overexpression of miRNAs implicated in inflammatory (miR-223, -146b), proliferative, and differentiative phases (miR-126, -199a) of the healing process (iv) Enhanced muscle regeneration process	[89]
Human	Anoxia (0.1% O_2_), hypoxia (5% O_2_)	*α*MEM +5 g/L glucose	(i) CM from anoxic conditions enhanced chemotactic and proangiogenic properties and reduced inflammatory mediator content (ii) Enhanced expression of VEGF-A, VEGF-C, IL-8, RANTES, and monocyte chemoattractant protein 1	[86]
Human adipose tissue	Hypoxia (5% O_2_)	RKCM	(i) Promoted antiapoptotic effects (ii) Higher levels of GM-CSF, VEGF, IL-6, and IGF-1 (iii) Lower levels of TGF-*β*1	[92]
Human umbilical cord Wharton jelly	Hypoxia (5% O_2_)	PPRF-msc6	(i) Increased secretion profile (ii) Upregulated thymosin-beta and EF-2 significantly (iii) Enhanced neuroregulatory secretome profile	[90]
Human bone marrow	Hypoxia (1% O_2_)	DMEM + 10% FBS + 2 mM L-glutamine	(i) Upregulated protein level of vimentin, fibronectin, and N-cadherin (ii) Enhanced stemness genes Oct4, Nanog, Sall4, and Klf4 (iii) Higher levels of osteocalcin and osteopontin (iv) Reduced levels of COL2A1, COMP, and aggrecan (v) Lower expression of adipsin, FASN, and FABP4 (vi) Upregulated IGFs, VEGF, EGF, GCSF, GM-CSF, TGF-*β*1, and TGF-*β*2	[93]
Human bone marrow	Hypoxia (1% O_2_) with serum starvation	Opti-MEM + 1% L-glutamine	(i) Significant increases in rate-limiting proteins of glycolysis and the NRF2/glutathione pathway (ii) Upregulated angiogenic associated pathways of PDGF, EGF, and FGF (iii) Microvesicle secretion decreased, exosome secretion substantially increased	[88]
Murine bone marrow	Repeated cycles of anoxia	StemPro MSC SFM	(i) miR-11, miR-22, miR-24, miR-199a-3p, and miR-210 upregulated in exosomes	[91]
Human bone marrow	Hypoxia for 30, 60, or 90 min	Unknown	(i) The 60 min group had the greatest protective effect on endotoxin-induced acute lung injury model	[94]

Mechanical stimuli				
Human bone marrow	TGF-*β*1 stimulation (1 ng/mL) or mechanical load (multiaxial shear and compression) in fibrin-poly(ester-urethane) scaffolds	*α*MEM + 10% FBS + 5 ng/mL bFGF	(i) TGF-*β*1 stimulation and load had distinct effects, both enhanced chondrogenic profile compared to control (ii) Nitrite content in media higher in loaded groups (iii) TGF-*β*1 enhanced expression of leptin, leptin receptor, and MDC (iv) Load enhanced expression of uPAR, LAP, MIP3*α*, angiogenin, ALCAM, angiopoietin 2, osteoprotegerin, and DR6; reduced expression of GRO (v) Both TGF-*β*1 and load enhanced the expression of BLC, MCP3, MIF, VEGF, MMP13, and PDGFaa	[100]
Human bone marrow	Computer-controlled bioreactors, on Cytodex 3 microcarriers (2 g/L)	PPRF-msc6	(i) Enhanced the neuroregulatory profile of secretome (ii) Increased the secretion of Cys C, GDN, Gal-1, and PEDF (iii) Upregulation of miR-16 (iv) Number of CNS regulators only detected in CM of bioreactor cultured MSCs (v) Upregulation of classical trophic factors BDNF, VEGF, and IGF-1	[16]
Human bone marrow	Bioreactors	DMEM + 10% FBS	(i) Enhanced angiogenesis by CM from mechanically stimulated MSCs via FGFR and VEGFR signalling cascades (ii) Enrichment of MMP-2, TGF-*β*1, and bFGF	[102]
Human	PAM hydrogels of various rigidity	DMEM-low glucose + 10% FBS	(i) VEGF, angiogenin, and IGF upregulated with increasing elastic modulus (ii) EGF, IL-6, and IL-8 were not stiffness-dependent	[103]
Adipose tissue	Fibrous scaffolds of variously aligned fibers	*α*-MEM + 10% FBS	(i) Higher levels of anti-inflammatory and proangiogenic cytokines were produced from cells seeded on electrospun scaffolds (ii) CM from scaffold cultures accelerated wound closure and macrophage recruitment in wound bed	[163]

Electromagnetic stimuli				
Equine adipose tissue	Static magnetic field (0.5 T)	DMEM/F12 + 10% FBS	(i) Reached doubling time earlier, colony-forming potential higher (ii) Considerable increase in the number of secreted microvesicles (iii) Release of BMP-2, VEGF, and p53 increased (iv) Reduced release of TNF-*α*	[113]

Biochemical stimuli				
Human bone marrow	IFN-*γ* and TNF-*α* stimulation	DMEM-low glucose	(i) Elevated secretion levels of IL-6, HGF, VEGF, and TGF-*β*	[119]
Murine bone marrow	IFN-*γ* and either TNF-*α*, IL-1*α*, or IL-1*β* stimulation	*α*-MEM+ 10% FBS + 2 mM glutamine	(i) Provoked the expression of CXCL-9 and CXCL-10 and inducible nitric oxide synthase	[118]
Human adipose tissue	TGF-*β*1 stimulation	DMEM + 0.1% BSA + 1% glutamine	(i) Upregulated secretion of PIGF, IGFBP-3, LIF, OSM, IL-4, IL-7, IL-13, CXCL9, CCL26, and OPN (ii) Downregulated secretion of CCL7, CCL11, CXCL6, OPG, IL-5, IL-10, CCL8, CXCL1, CXCL10, HGF, leptin, FGF-7, and GM-CSF	[123]
Adipose tissue	TNF-*α* stimulation	MesenPRO RS Basal Medium + 2 mM L-glutamine + MesenPRO RS Growth Supplement	(i) TNF-*α*-preconditioned ASCs secreted exosomes with elevated Wnt-3a content (ii) Enhanced proliferation and osteogenic differentiation in human primary osteoblastic cells	[120]
Human umbilical cord	LPS preconditioning	DMEM-low glucose + 10% FBS and sigma serum-free medium	(i) Improved regulatory abilities for macrophage polarization and resolution of chronic inflammation (ii) Unique expression of miR-let7b	[115]
Human adipose tissue	LPS preconditioning	DMEM-low glucose	(i) Enhanced mRNA expression of IL-6, TNF-*α*, HGF, and VEGF (ii) Enhanced liver regeneration in partially hepatectomized mice	[116]
Human adipose tissue	H_2_O_2_ stimulation	*α*-MEM + 10% exo-free FBS	(i) Exosomes that had been H_2_O_2_-stimulated enhanced skin flap recovery and capillary density	[121]

## Abbreviations

Akt: Protein kinase B
ALCAM: Activated leukocyte cell adhesion molecule
*α*MEM: Alpha minimal essential medium
ALP: Alkaline phosphatase
ATP: Adenosine triphosphate
bFGF: Basic fibroblast growth factor
BLC: B lymphocyte chemoattractant
BMP-2: Bone morphogenetic protein-2
CCL: Chemokine (C-C motif) ligand
CCM: Complete culture medium
CD: Cluster of differentiation
CKD: Chronic kidney disease
CM: Conditioned medium
CNS: Central nervous system
COMP: Cartilage oligomeric matrix protein
CTX: Cardiotoxin
CXCL: Chemokine (C-X-C motif) ligand
Cys: Cystatin
DMEM: Dulbecco's modified Eagle's medium
DR6: Death receptor 6
ECM: Extracellular matrix
EGF: Epidermal growth factor
EGFR: Epidermal growth factor receptor
EMF: Electromagnetic field
EV: Extracellular vesicle
FABP: Fatty acid binding protein
FASN: Fatty acid synthase
FBS: Fetal bovine serum
FGF: Fibroblast growth factor
Gal: Galactosidase
GAP: Growth-associated protein
GC: Glucocorticoid
G-CSF: Granulocyte colony-stimulating factor
GDN: Glia-derived nexin
GM-CSF: Granulocyte macrophage colony-stimulating factor
GRO: Growth-related oncogene
GvHD: Graft versus host disease
H_2_O_2_: Hydrogen peroxide
HGF: Hepatocyte growth factor
HIF: Hypoxia-inducible factor
HPL: Human platelet lysate
HUVEC: Human umbilical vein endothelial cell
IFN: Interferon
IGF: Insulin-like growth factor
IGFBP: Insulin-like growth factor binding protein
IGFR: Insulin-like growth factor receptor
IL: Interleukin
LAP: Latency-associated peptide
LIF: Leukemia inhibitory factor
LPS: Lipopolysaccharide
MCP: Monocyte chemotactic protein
MDC: Macrophage-derived chemokine
MIF: Macrophage migration inhibitory factor
MIP: Macrophage inflammatory protein
MMP: Matrix metalloproteinase
MS: Multiple sclerosis
MSC: Mesenchymal stem cell
MVB: Multivesicular body
NADH: Nicotinamide adenine dinucleotide
NO: Nitric oxide
OPG: Osteoprotegerin
OPN: Osteopontin
OSM: Oncostatin M
P53: Tumor suppressor 53
PAH: Pulmonary arterial hypertension
PDGF: Platelet-derived growth factor
PEDF: Pigment epithelium-derived factor
PIGF: Placental growth factor
RANTES: Regulated on activation, normal T cell expressed and secreted
RGC: Retinal ganglion cell
ROS: Reactive oxygen species
RNA: Ribonucleic acid
RT-qPCR: Quantitative reverse transcription
SD: Sprague Dawley
SDF: Stromal cell-derived factor
SF: Serum-free
SFM: Serum-free media
STC: Stanniocalcin
STZ: Streptozocin
T1DM: Type 1 diabetes mellitus
T2DM: Type 2 diabetes mellitus
TB: Thymosin beta
TGF: Transforming growth factor
Th: T helper
TNF: Tumor necrosis factor
TSG: Tumor necrosis factor-inducible gene protein
uPAR: Urokinase-type plasminogen activator receptor
VEGF: Vascular endothelial growth factor
XF: Xeno-free.
